# Elevated Th22 as well as Th17 cells associated with therapeutic outcome and clinical stage are potential targets in patients with multiple myeloma

**DOI:** 10.18632/oncotarget.4641

**Published:** 2015-06-25

**Authors:** Min Wang, Ping Chen, Yan Jia, Na He, Daqi Li, Chunyan Ji, Daoxin Ma

**Affiliations:** ^1^ Department of Hematology, Qilu Hospital, Shandong University, Jinan, China; ^2^ Department of Hematology, Jinan Central Hospital, Affiliated to Shandong University, Jinan, China

**Keywords:** multiple myeloma, Th22, Th17, Th1

## Abstract

T helper (Th) cell imbalance plays important roles in tumor development and their effects in Multiple myeloma (MM) remain unclear. In the present study, we investigated the levels and clinical significance of Th22, Th17 and Th1 cells in patients with MM. Th subsets were examined by flow cytometry. Plasma IL-22, IL-17A and IFN-γ concentrations were measured by ELISA. AHR and RORC mRNA expression was examined by RT-PCR. Here, we found that the frequency of Th22 cells was significantly elevated in peripheral blood (PB) and bone marrow (BM) of newly-diagnosed MM patients, and recovered in complete remission patients after chemotherapy. The circulating Th17 cells accompanied by IL-17A levels were also up-regulated in MM patients and decreased after remission. We also found that there was a significantly positive correlation between Th22 and Th17 cells in MM patients. Moreover, the frequencies of Th22 and Th17 cells were higher in stage III than in stage I+II of MM. Our data demonstrated that Th22 and Th17 cells might be important therapeutic targets in multiple myeloma and could facilitate the effect of antitumor immunotherapy.

## INTRODUCTION

Multiple myeloma (MM) is characterized by the clonal expansion of plasma cells and the systemic damage caused by monoclonal protein [1]. The pathogenesis of MM is complex and heterogeneous, which involves a series of genetic and immunological alterations. Many abnormalities of T cell number or function have been observed in patients with MM. However, the precise mechanisms and biologic basis for these abnormalities remain unclear [2, 3].

Accumulating evidences have demonstrated that T helper (Th) cells play critical roles in the development and progression of inflammatory diseases, autoimmune diseases and malignant tumors. Th17, a novel CD4 subset, which expresses the lineage-specific transcription factor retinoic acid receptor-related orphan receptor C (RORC), was identified on the basis of producing IL-17A but not IFN-γ [4]. Th22 is a more recently identified human inflammatory Th subset, which is characterized by abundant secretion of IL-22 and TNF-α, but not IFN-γ, IL-4 or IL-17A [5]. Aryl hydrocarbon receptor (AHR) has been considered to be the key transcription factor of Th22 subset and is involved in IL-22 expression [6]. Both Th17 and Th22 are proved to be involved in the pathophysiology of autoimmunity and tumorigenesis. Before the discovery of Th17 and Th22 cells, inflammatory CD4^+^ T cell studies in diseases mainly focused on Th1 cells, which produce IFN-γ as their signature cytokine [7].

Abundant evidences about Th17 have focused on patients with autoimmune diseases while there are very few studies on cancer patients, and the specific role of Th17 in cancer is debatable [8]. In the case of MM, the previous researches associated with Th17 and IL-17 were contradictory. Prabhala et al observed significantly increased Th17 and IL-17A in MM which promoted MM cell growth as well as suppressed immune responses [9]. Alexandrakis et al [10] and Shen CL et al [11] also demonstrated increasing levels of Th17 and IL-17 in association with higher disease stage, and suggested that IL-17 plays a role in promoting angiogenesis and disease progression in MM. However, Braga et al. considered that Th17 may promote immune responses in MM patients [2]. As for the conflicting results, another research explained that the suppressing or promoting effect of higher IL-17 in MM may be due to the balance between IL-17A and IL-17E [12]. Therefore, the mechanisms of Th17 immune abnormalities in MM remain elusive.

Th22 cells have been shown to be important in the pathogenesis of many autoimmunity diseases. Our and other studies demonstrated that Th22 were elevated in patients with immune thrombocytopenia (ITP) [13], myelodysplastic syndrome (MDS) [14], ankylosing spondylitis (AS) and rheumatoid arthritis (RA) [15]. However, up to now, there is no data available with regard to the roles of Th22 and related cytokines in the pathophysiology of MM. In addition, the relationship of Th22 with Th17 or Th1 in MM patients is still unclear and remains to be clarified.

Therefore, in order to identify and describe the possible roles of Th22 and Th17 subsets in the pathogenesis of MM, we investigated the proportions of pure Th17 (CD4^+^ IFN-γ^−^IL-17A^+^IL-22^−^ T cells), pure Th22 (CD4^+^IFN-γ^−^IL-22^+^IL-17A^−^ T cells) as well as Th1 cells (CD4^+^IFN-γ^+^ T cells) in the peripheral blood and bone marrow by flow cytometry, concentrations of plasma IL-17A, IL-22 and IFN-γ by enzyme-linked immunosorbent assay (ELISA) along with the mRNA expression levels of RORC, AHR in Chinese patients with MM. We evaluated their clinical relevance and relationship with therapy. Furthermore, their correlation with disease severity was also assayed in this study.

## RESULTS

### Both peripheral and bone marrow Th22 cells were elevated in newly-diagnosed MM patients and recovered after complete remission

**Figure 1:**
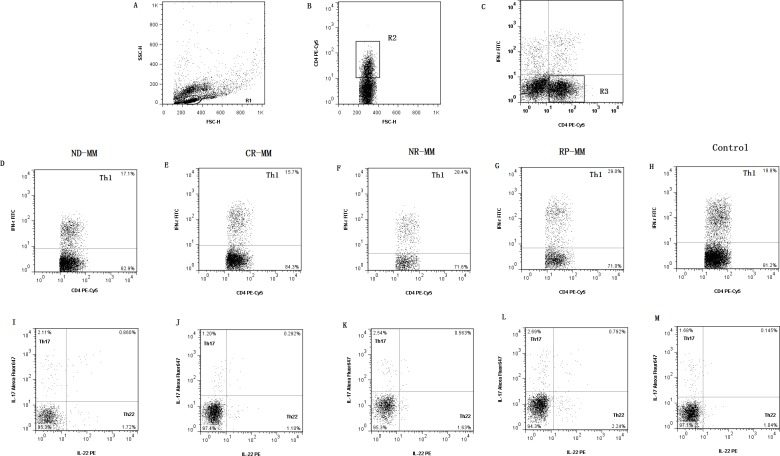
Dot plots of Th1, Th17, Th22 and IL-17/IL-22 double-positive T cells (**A**) Lymphocytes were gated in R1. (**B**) CD4^+^ T cells were gated in R2. (**C**) CD4^+^ IFN-γ^−^ T subsets were gated in R3 and R3 were used for the analysis of I-M. (**D, E, F, G, H**) Representative FACS dot plots of Th1 (CD4^+^IFN-γ^+^) cells as a proportion of CD4^+^ T cells from ND, CR, NR, RP MM patients and healthy controls. (**I, J, K, L, M**) Representative IL-22 and IL-17 expression in CD4^+^ IFN-γ^−^ T subsets from ND, CR, NR, RP MM patients and healthy controls. The percentages of circulating Th22 (CD4^+^IFN-γ^−^IL17^−^IL-22^+^), Th17 (CD4^+^IFN-γ^−^IL-22^−^IL17^+^) and IL-17/IL-22 double-positive (CD4^+^IFN-γ^−^IL17^+^IL-22^+^) T cells were shown in the lower right, upper right and upper left quadrants, respectively.

**Figure 2:**
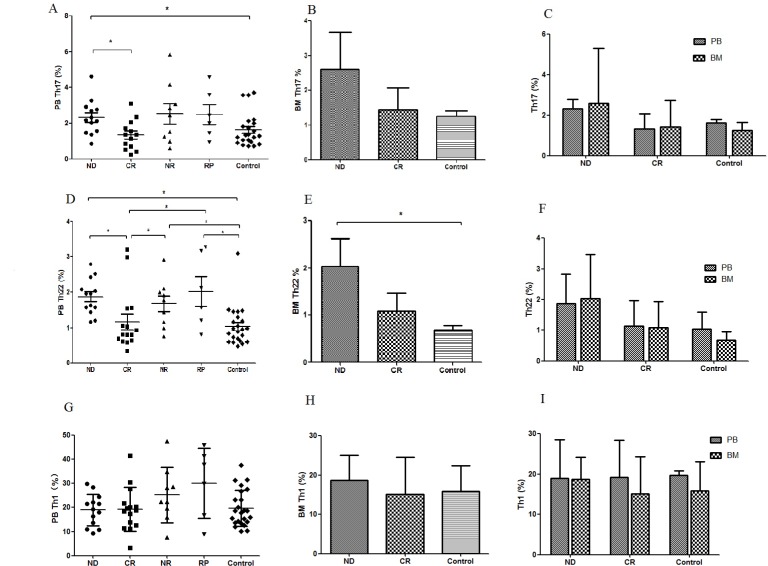
Results of Thl7, Th22 and Thl (**A**) Circulating Thl7 cells were significantly increased in ND MM patients compared to CR MM patients or healthy controls. (**B**) There was no significant difference between ND and CR patients or healthy controls. (**C**) No significant difference ofThl7 cells was found between PB and BM. (**D**) Circulating percentages ofTh22 cells were significantly higher in ND, NR or RP patients compared with healthy controls or CR patients. (**E**) The frequency ofBM Th22 cells in ND patients was proudly higher than that in controls. (**F**) There was no difference of Th22 cells between PB and BM in MM patients or healthy controls. (**G**) No statistical difference of circulating Th1 cells was found between each group. (**H**) There was no statistical difference of BM Thl cells between each group. (**I**) Thl cells in PB and BM were not statistically different in MM patients or healthy controls.

The typical dot plots of Th22 cells in representative MM patients and healthy controls were shown in Fig. 1I-1M. Compared with healthy controls (1.04±0.61%), the frequency of peripheral Th22 was observably elevated in ND patients (1.87±0.96%, P=0.048), NR patients (1.68±0.49%, P=0.025) or RP patients (2.03±0.34%, P=0.011). Furthermore, a significant decrease of peripheral Th22 was seen in CR patients (1.17±1.29%) after chemotherapy compared with ND (P=0.003), NR (P=0.046) or RP (P=0.032) patients (Fig. 2D). Meanwhile, we further analyzed the frequency of Th22 cells in BM and found there was a remarkable increase in ND patients (2.04±1.43%, P=0.022) compared with those in controls (0.68±0.27%). The frequency of Th22 in CR patients (1.08±0.86%) was lower than that in ND patients but still higher than controls (Fig. 2E). No significant difference of Th22 was found between PB and BM in MM patients or controls (Fig. 2F).

**Figure 3:**
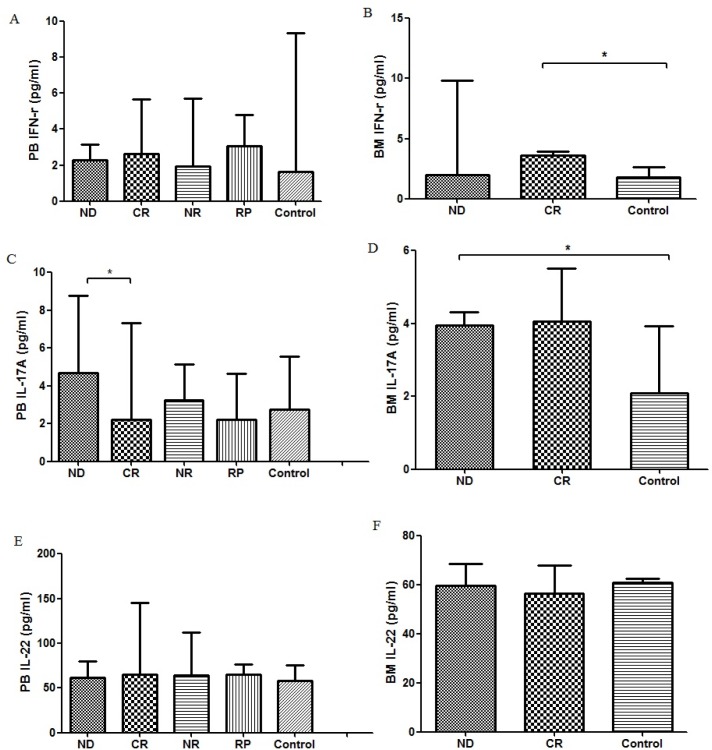
(**A**) Concentration of PB IFN-γ in MM patients was not significantly different with that in healthy controls (**B**) Concentration of BM IFN-γ in CR MM patients was markedly higher than that in healthy controls. (**C**) Concentration of PB IL-17A in CR MM patients was significantly decreased than that in ND patients. (**D**) Concentration of BM IL-17 in ND patients was significantly higher than that in healthy controls. (**E**) There was no significant difference of PB IL-22 between each group. (**F**) Concentrations from ND, CR MM patients and healthy controls. There was no significant difference of IL-22 in BM between each group.

As for PB plasma IL-22, there was no significant difference between each group (ND: median, 61.34, range: 50.31-79.49 pg/ml; CR: median, 65.23, range: 49.66-145.34 pg/ml; NR: median, 63.93, range: 49.01-112.46 pg/ml; RP: median, 65.23, range: 56.15-76.25 pg/ml; controls: median, 58.10, range: 47.71-75.60 pg/ml) (Fig. 3E and Table 1). Similarly, the levels of BM plasma IL-22 in ND, CR MM patients and controls were not significantly different (ND: median, 59.72, range: 49.00-67.18 pg/ml, P>0.05; CR: median, 56.47, range: 53.56-67.82 pg/ml, P>0.05; control: median, 61.02, range: 53.56-62.64 pg/ml) (Fig. 3F).

**Table 1:** Plasma concentrations of Th1/Th17/Th22-associated cytokines in patients with multiple myeloma

PB cytokine (pg/ml)		ND	CR	NR	RP	Control	Statistical significance
**IL-22**	median	61.34	65.23	63.93	65.23	58.10	**NS**
	range	50.31-79.49	49.66-145.34	49.01-112.46	56.15-76.25	47.71-75.60	
**IL-17A**	median	4.67	2.22	3.26	2.20	2.73	
	range	0.99-8.78	0.86-7.32	1.04-5.14	0.90-4.63	0.95-5.57	
**IFN-γ**	median	2.27	2.62	2.03	3.08	1.66	**NS**
	range	1.33-3.13	1.35-5.66	1.26-5.71	1.08-4.79	1.05-9.34	

NS: no significance

No correlation was identified between peripheral Th22 frequency and circulating IL-22 level.

### Circulating Th17 cells accompanied by IL-17A levels were up-regulated in MM patients and decreased after remission

Peripheral Th17 cells were markedly increased in patients with ND MM (2.32±0.45%) than those in control group (1.63±0.16%, P=0.048). Additionally, the percentages of Th17 cells in NR patients (2.52±1.58%) or RP patients (2.49±2.22%) were also higher compared to controls, but did not reach statistical difference (P>0.05). Moreover, the frequency of Th17 cells in CR patients (1.34±0.73%, P=0.013) was significantly decreased compared with ND patients (Fig. 2A). Meanwhile, we also evaluated the frequency of Th17 cells in the BM microenvironment of MM patients, and the results showed that Th17 percentage in ND patients was higher (2.6 ± 2.7%) than that in CR patients (1.43±1.30%) or in control groups (1.25±0.40%), but did not reach statistical difference (P>0.05) (Fig. 2B). Additionally, we compared Th17 cells between PB and BM in MM patients or controls, and no statistical difference was found (Fig. 2C).

The levels of IL-17A in PB and BM were measured by ELISA. Compared with ND patients (median, 4.67; range, 0.99-8.78 pg/ml), PB plasma IL-17A level was significantly decreased in CR patients (median, 2.22; range, 0.86–7.32 pg/ml, P=0.004). Although the levels of PB plasma IL-17A in NR patients (median, 3.26; range, 1.04–5.14 pg/ml), RP patients (median, 2.20; range, 0.90–4.63 pg/ml) and controls (median, 2.73; range, 0.95–5.57 pg/ml) were lower than those in ND patients, no statistical significance was observed (Fig. 3C and Table 1). As for the BM plasma IL-17 level, a statistical increase was found in ND patients compared with healthy controls (Fig. 3D).

However, no correlation was found between Th17 cells and plasma levels of IL-17A both in PB and BM of MM patients.

### Increased IL-17 and IL-22 double positive CD4+ T cells in MM patients

In these experiments, we also examined the frequencies of CD4^+^ IFN-γ^−^IL-17A^+^IL-22^+^ T cells in MM patients. The percentage of circulating CD4^+^IFN-γ^−^IL-17A^+^IL-22^+^ T cell was significantly increased in ND (0.40±0.20%), NR (0.52±0.04%) and RP (0.77±0.58%) patients compared with healthy controls (0.13±0.04%). In addition, in CR patients (0.24±0.09%), the percentage of this double-positive cells was markedly decreased than those in ND patients but still higher than those in healthy controls (Fig. 1I-1M).

### Th1 cells and INF-γ in MM patients

For peripheral Th1 cells, as shown in Fig. 1D-1H and Fig. 2G, no significant difference was observed between each group (ND: 18.96±9.55%, P>0.05; CR: 19.23±9.23%, P>0.05; NR: 25.27±19.35%, P>0.05; RP: 30.09±17.36%; controls: 19.66±1.20%). This finding was similar to Th1 results in BM (ND: 18.74±5.48%, P>0.05; CR: 15.14±9.14%, P>0.05; controls: 15.92±7.11%) (Fig. 2H). Furthermore, there was no difference between BM and PB in MM patients and healthy controls (Fig. 2I).

Compared with healthy controls (median, 1.66; range, 1.05-9.34 pg/ml), no significant change of PB plasma IFN-γ level was found in ND patients (median, 2.27; range, 1.33-3.13 pg/ml; P>0.05), CR patients (median, 2.62, range, 1.35-5.66 pg/ml; P>0.05), NR patients(median, 2.03, range, 1.26-5.71 pg/ml; P>0.05) and RP patients(median, 3.08, range, 1.08-4.79 pg/ml; P>0.05). However, as for the BM plasma IFN-γ level, a statistical increase was only found in CR patients compared with healthy controls (Fig. 3A, 3B and Table 1).

### Expression levels of RORC and AHR in MM patients

Though the expression level of RORC or AHR was higher in ND MM patients (median=0.10, range, 0.01-0.25; median=0.48, range, 0.09-1.21, resp) compared to control group (median, 0.08; range, 0.003-0.43; median, 0.44; range, 0.11-0.99, resp), no statistical difference was found. There was no significant change between other groups.

### Th22 showed positive correlation with Th17 cells in MM patients

**Figure 4:**
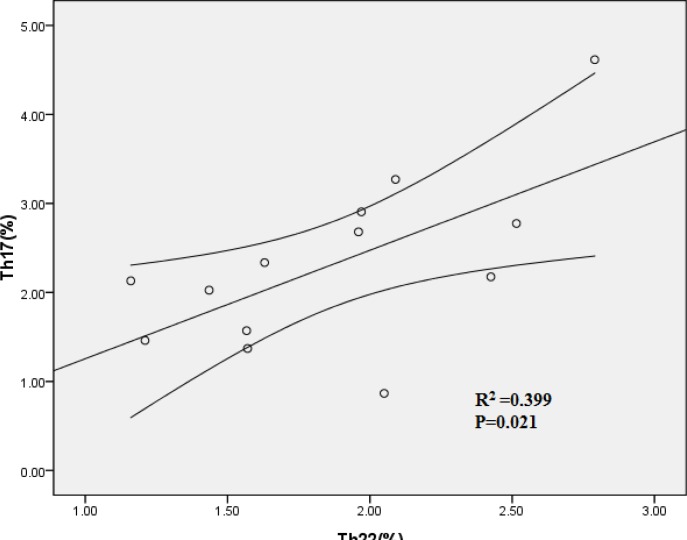
Positive correlation was found between PB Th22 and Th17 cells in ND patients

Th22 showed significantly positive correlation with Th17 in PB of ND MM patients (R=0.399, P=0.021) (Fig. 4). However, Th1 cells failed to show significant correlation with Th22 or Th17 in MM patients.

### The correlations of Th1/Th17/Th22 cell with the clinicopathological characteristics of patients with MM

The relationships between the frequency of Th1/Th17/Th22 cells and various clinicopathological characteristics across the study population are shown in Table 2. There was no relationship between the proportion of Th1/Th17/Th22 cells and patient age or gender. There is also no relationship between Th1/Th17/Th22 cells and MM subtype.

**Table 2:** The correlations of Th1/Th17/Th22 frequencies with clinicopathological characteristics of MM patients

Characteristic	N	Th1%	Th17%	Th22%
**Gender**				
Male	36	21.35±4.07	2.36±1.82	1.50±0.67
Female	19	23.15±5.62	2.04±1.64	2.17±1.96
P value		0.6	0.606	0.744
**Age/year**				
<60	37	22.71±18.88	2.25±1.92	1.60±1.41
≥60	18	21.20±16.79	2.23±1.32	1.89±0.79
P value		0.639	0.979	0.464
**Type**				
IgG	26	21.99±24.64	2.23±2.09	1.63±0.81
IgA	12	20.60±7.74	2.21±1.45	1.64±0.85
IgD	17	22.42±15.17	1.31±1.21	1.65±0.62
P value		0.932	0.638	0.999

### Th22 as well as Th17 cells up-regulated with the increase of MM clinical stage

**Figure 5:**
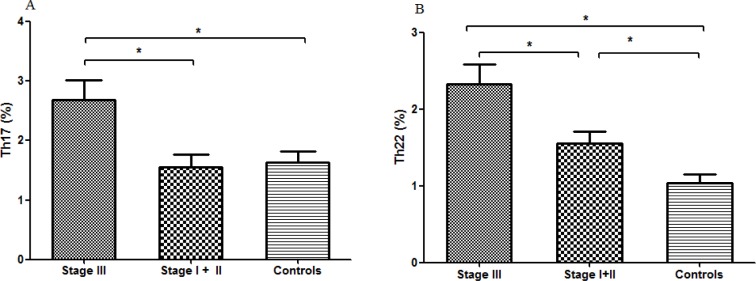
Circulating Th17 and Th22 cells of stage III, stage I+II MM patients and healthy controls (**A**) Circulating Th17 cells remained significantly higher in stage III patients compared with that in stage I+II patients or healthy controls. (**B**) Circulating percentages of Th22 cells were significantly higher in stage III patients compared with stage I+II patients and healthy controls.

We further analyzed the relationship between Th1/Th17/Th22 cells and tumor stage. As shown in Fig. 5A, peripheral Th17 significantly increased in stage III patients (2.69±0.94%) compared with stage I+II patients (1.55±0.47%, P=0.015) and controls (1.63±0.16%, P=0.01), while no significant difference was seen between stage I+II and controls. As for the correlations between peripheral Th22 frequency and disease stage, the frequency of Th22 in stage III patients (2.33±0.72%) was markedly higher than that in stage I+II MM patients (1.55±0.55%, P=0.025) and controls (1.04±0.56, P=0.001). Th22 frequency in stage I+II patients was also higher than that in controls (P=0.027) (Fig. 5B).

## DISCUSSION

Multiple myeloma is characterized by the uncontrolled proliferation of monoclonal plasma cells in the bone marrow. Although bortezomib combined with chemotherapy and transplantation is relatively effective treatment, MM continues to be an incurable disease with fatal outcome for the majority of patients at advanced stages. Therefore, exploration of novel therapeutic modalities should be pursued [16].

The host immune system dysfunction has a pivotal role for plasma cells and is responsible for some clinical manifestations of MM [17, 18]. Recently, the aberrance of T helper cells has been described to be involved in the pathogenesis of MM patients. However, the biologic basis of this dysfunction remains ill-defined. So in this study, we examined the frequencies of Th22, Th17 and Th1 cells and the concentrations of plasma IL-22, IL-17A and IFN-γ along with specific transcription factor expression in MM patients and healthy controls.

Compared with the well-known Th1, Th2 and Th17, Th22 is a newly-identified Th subset with a specific phenotype and distinct function. IL-22 was also secreted by activated Th1, Th17, NK cells, NKT cells and lymphoid tissue inducer (LTi) cells [19]. Th22 and IL-22 were recently reported to play important roles in chronic inflammation and tumorigenesis [19]. So far, there is little data with regard to the Th22 cells and related cytokines in the onset and development of MM. Recently, Giulia et al. found that IL-22^+^IL-17^−^IL-13^+^ T cells increased in PB and BM of stage III and RP MM patients compared with asymptomatic or stage I/II patients and controls. They also found a fraction of MM cell lines and primary tumors aberrantly expressed IL-22RA1 and demonstrated that IL-22 induced STAT-3 phosphorylation, cell growth, and resistance to drug. They concluded that Th22 cells increase in poor prognosis MM and promote tumor cell growth and survival [20]. In the present study, we identified a notably expanded Th22 population in PB and BM microenvironment of MM patients compared with healthy controls. Moreover, the frequency of Th22 cells in MM patients who achieved complete remission was significantly reduced after chemotherapy, which indicated that chemotherapy may partly ameliorate this Th22 turmoil. These suggested that measurement of Th22 frequency may be useful in evaluating therapeutic effect. Similarly, our results also demonstrated that Th22 percentage in stage III was significantly higher than that in stage I+II patients, while Th22 percentage in stage I+II patients was also higher that in healthy controls. These findings suggest that there was a significant relationship between Th22 and clinical tumor stage, and further prove the potential pathogenesis of Th22 in MM.

The expression level of cytokine IL-22 and specific transcription factor AHR has not been investigated in MM until today. IL-22 belongs to the IL-10 cytokine family and is a less well-defined tissue-instructing cytokine [21]. However, inconsistent with the Th22 in MM patients, our results demonstrated that IL-22 level either in PB or BM of MM patients was comparable with that of healthy controls, and no correlation with peripheral Th22 was found. This phenomenon may indicate that the unilateral increased frequency of Th22 contributes little to the IL-22 production on account of IL-22 is not exclusively produced by Th22 cells. While, in accordance, the mRNA expression of AHR was elevated in MM patients compared with healthy controls, notwithstanding there was no significant difference, this may be due to the limited sample numbers. Obviously, further researches of larger sample size are needed to explain the specific roles of Th22 cells in MM patients.

By far, the impact of Th17 and associated cytokines on cancer development is still under debate. Although multiple reports indicate that Th17 can promote anticancer immunity, others have argued that these cells may exhibit tumor-promoting properties [8]. Currently, there are a few reports indicate an increase of Th17 cells in the PB and BM of MM patients, which are in accordance with our findings [10, 12, 22]. The increase of Th17 in MM patients may be caused by the increased proinflammatory cytokines, and eventually lead to Th17 polarization in MM milieu. Th17 expansion along with increased related cytokines suppressed immune responses, protected myeloma cells from CTL attack, and promoted their survival and growth [17]. In our study, Th17 increased in ND, NR or RP MM patients, and reduced after achieving complete remission. Similar to Th22, Th17 was also correlated with tumor stage, which was consistent with Shen's study [11]. These suggested that measurement of Th17 frequency might be related to tumor burden and may be useful in evaluating therapeutic effect.

IL-17 cytokines, IL-17A to IL-17F, are emerging as critical players in host defense responses and inflammatory diseases. Th17 is the predominant cell-type to produce IL-17A and IL-17F, while there are still other multiple cell types expressing IL-17A [23]. In our study, in line with the increased Th17 cells, plasma IL-17A was elevated in ND patients and reduced after achieving CR. The increased IL-17A along with elevated Th17 in the current study may contribute to the pathogenesis of MM. As for RORC expression, no statistical difference was found between MM patients and controls, which may be because we detected the expression of RORC in the total lymphocytes rather than CD4^+^ T cells.

In our study, there existed a positive correlation between peripheral Th22 and Th17 subset in ND MM patients, implying that differentiation of Th22 and Th17 may be induced in an influential manner in MM. This co-elevated frequency of Th22 and Th17 suggested that these two Th subsets may play a synergistic role in leading to a dramatic increase in the over-exuberant inflammatory immune reaction in MM patients.

Numerous evidences suggest Th1 cells have been linked to the development of autoimmune inflammatory processes [14]. Regarding the specific roles of Th1 and related cytokines IFN-γ in MM, the results were controversial. One study reported decreased Th1 responses with IFN-γ production [24, 25], while another report found that the levels of Th1 and IFN-γ were significantly higher in ND MM patients than controls [22]. However, our results were in disagreement with those above observation of decreased or increased Th1 responses in MM patients. In contrast to the results of Th22 and Th17, no statistical difference was shown in Th1 between patients with MM and controls. Meanwhile, statistical correlation between Th1 and Th22 or Th17 was not found in this study.

In conclusion, our study revealed for the first time that the proportion of Th22 and Th17 was increased in patients with MM. Moreover, there exited a significantly positive correlation between Th22 and Th17. In addition, there was a significant relationship between the proportion of Th22 or Th17 cells and clinical tumor stage. Taken together, our results indicate that Th22 and Th17 cells may represent important potential immunotherapeutic targets in MM. Further researches including *in vitro* or animal model studies are needed to understand their detailed roles in the pathobiology of MM.

## MATERIALS AND METHODS

### Subjects and ethics statement

A total of 55 MM patients were enrolled from Hematology Department of Qilu Hospital, Shandong University between July 2013 and August 2014. The diagnosis of multiple myeloma was based on the International Myeloma Working Group criteria [26]. The patients were classified according to the international staging system for multiple myeloma [27]. The patients were divided into four groups: 16 newly-diagnosed (ND) patients (3 females and 13 males; median age, 63; range, 40–80 years), 20 complete remission (CR) patients (10 females and 10 males; median age, 65; range, 37–72 years), 10 non-remission (NR) patients (4 females and 6 males; median age, 51; range, 40–80 years) and 9 relapsed-refractory (RP) patients (2 females and 7 males; media age, 57; range, 42–74 years). Twenty-three healthy PB donors (9 females and 14 males; median age, 43; range, 32-59 years) were also included in the study. Ten hematological normal BM transplant donors (3 females, 7 males; median age, 45; range, 36-55 years) were used as BM controls. In our study, CR is defined as confirmed negative immunofixation of serum and urine, and disappearance of any soft tissue plasmacytomas and plasma cells ≤5% in bone marrow. The criteria to define CR also include normal free light chain (FLC). This study was approved by the Medical Ethical Committee of Qilu Hospital, Shandong University, China. Informed consent was obtained from all patients before enrollment in the study in accordance with the Declaration of Helsinki.

### Treatment regimen

Newly-diagnosed MM patients were treated with 21-day cycles of bortezomib/thalidomide/dexamethasone (BTD) chemotherapy regimens: bortezomib 1.3mg/m^2^ on days 1, 4, 8, and 11 by subcutaneous injection, thalidomide 200mg/day given orally and dexamethasone 20 mg on days 1, 2, 4, 5, 8, 9, 11, and 12 via intravenous infusion.

### Flow cytometric analysis of Th22, Th17 and Th1 cells

Intracellular cytokines were studied by flow cytometry to reflex the cytokine-producing cells. Briefly, heparinized whole blood (100 μl) with an equal volume of Roswell Park Memorial Institute (RPMI)-1640 medium was incubated for 4 h at 37 °C in 5% CO2 in the presence of 2.5 ng/ml of phorbol myristate acetate (PMA), 1mg/ml of ionomycin, and 1.7mg/ml of monensin (all from Alexis Biochemicals, San Diego, CA, USA). PMA and ionomycin are pharmacologic T-cell activating agents that mimic signals generated by the T-cell receptor (TCR) complex and have the advantage of stimulating T cells of any antigen specificity. Monensin is used to block the intracellular transport mechanisms, thereby leading to an accumulation of cytokines in the cells. After incubation, the cells were stained with PE-CY5 conjugated anti-human CD4 monoclonal antibody at room temperature in the dark for 20 min. The cells were next stained with FITC-conjugated anti-IFN-γ, Alexa Fluor 647 conjugated anti-IL-17A and PE-conjugated anti-IL22 monoclonal antibodies after fixation and permeabilization. All the antibodies were purchased from eBioscience, San Diego, CA, USA. Isotype controls were given to enable correct compensation and confirm antibody specificity. Fix-Perm reagents were from Invitrogen (Carlsbad, CA, USA). All samples were washed and collected using BD FACS Calibur Flow Cytometer. Data were analyzed with FlowJo 7.6.2. For analysis, we first gated CD4^+^ lymphocytes, then analyzed the proportion of pure Th17 cells (CD4^+^ IFN-γ^−^IL-17A^+^IL-22^−^ T cells), pure Th22 cells (CD4^+^IFN-γ^−^IL-22^+^ IL-17A^−^ T cells) as well as Th1 cells (CD4^+^IFN-γ^+^ T cells) in CD4+ lymphocytes.

### Determination of the expression of RORC and AHR

The total RNA was extracted with Trizol (Invitrogen, Carlsbad, CA, USA) according to the manufacturer's instructions. Approximately, 1 μg of total RNA from each sample was used to synthesize cDNA with Prime Script RT reagent Kit (Takara Bio Inc., Dalian, China). Reverse transcription reaction was done at 37 °C for 15 min, followed by 85 °C for 5 s. Real-time quantitative PCR was conducted using an ABI Prism 7500 Real-time PCR system (Applied Biosystems, Foster City, CA, USA) in accordance to the manufacturer's instructions. The real-time PCR contained, in a final volume of 10 μl, 5 μl of 2× SYBR Green Real-time PCR Master Mix, 1 μl of cDNA, 3.2 μl of ddH2O and 0.4 μl of the forward and reverse primers. The primers were shown as follows: RORC Forward 5′-CAA TGG AAG TGG TGC TGG TTA G-3′, Reverse 5′-GGG AGT GGG AGA AGT CAA AGA T-3′; AHR Forward 5′-CAA ATC CTT CCA AGC GGC ATA-3′, Reverse 5′-CGC TGA GCC TAA GAA CTG AAA G-3′. All experiments were conducted in triplicate. The PCR products were analyzed by melt curve analysis and agarose gel electrophoresis to determine product size and to confirm that no by-product was formed. The results were expressed relative to the number of GAPDH transcripts used as an internal control. GAPDH was analyzed using the following primers: Forward 5′-GCT CTC TGC TCC TCC TGT TC-3′, Reverse 5′-GTT GAC TCC GAC CTT CAC CT-3′. Relative gene expression level (the amount of target, normalized to endogenous control gene) was calculated using the comparative Ct method formula 2^−ΔCt^.

### IL-22, IL-17A and IFN-γ concentration assay with ELISA

PB and BM were collected into heparin-anticoagulant vacutainer tubes. Plasma was obtained by centrifugation and stored at −80°C for determination of cytokines. The levels of IL-22 (Cat: BMS2047), IL-17A (Cat: BMS2017) and IFN-γ (Cat: BMS228) were measured by ELISA following the manufacturer's instructions (eBioscience, San Diego, CA). The lower detection limits were as follows: IFN-γ, 0.99pg/ml; IL-17A, 0.5 pg/ml; IL-22, 5 pg/ml.

### Statistical analysis

All of the data were given as mean ± SD or median (range). Statistical significance of Th22, Th17 and Th1 cells, and plasma IL-22, IL-17A as well as IFN-γ among MM patients in different groups was determined by ANOVA, and difference between two groups was determined by Newman-Keuls multiple comparison test (q test) unless the data were not normally distributed, in which case Kruskal-Wallis test (H test) and Nemenyi test were used. The Pearson or Spearman correlation test was used for correlation analysis depending on data distribution. All tests were performed by SPSS 17.0 system. P value less than 0.05 was considered statistically significant.
